# Goal-setting and personalization under the International Classification of Functioning, Disability, and Health framework: Community reintegration program for post-stroke patients

**DOI:** 10.3389/fresc.2023.1219662

**Published:** 2023-08-04

**Authors:** Mabel Ngai-Kiu Wong, Horace Tong, Mike Kwun-Ting Cheung, Yuk-Mun Ng, Huan-Ling Yuan, Bess Yin-Hung Lam, Siu Ngor Fu, Chetwyn Che Hin Chan

**Affiliations:** ^1^ Department of Rehabilitation Sciences, The Hong Kong Polytechnic University, Hong Kong SAR, China; ^2^ Department of Psychology, The Education University of Hong Kong, Hong Kong SAR, China; ^3^ Laboratory of Neuropsychology and Human Neuroscience, The University of Hong Kong, Hong Kong SAR, China; ^4^ Centre on Research and Advocacy, The Hong Kong Society of Rehabilitation, Hong Kong SAR, China; ^5^ Rehabilitation Division, The Hong Kong Society for Rehabilitation, Hong Kong SAR, China; ^6^ Department of Counselling and Psychology, Hong Kong Shue Yan University, Hong Kong SAR, China

**Keywords:** post-stroke rehabilitation, ICF, community reintegration, goal-setting, patient-therapist interaction, personalized treatment

## Abstract

**Background:**

Body functions and structures, activities, and participation are the core components in the International Classification of Functioning, Disability, and Health (ICF) to identify post-stroke patients' health conditions. The specification of health conditions enhances the outcomes of post-stroke rehabilitation.

**Purpose:**

This study aimed to explore the extent and the processes in an ICF-based post-stroke rehabilitation program (ICF-PSRP) that could enhance patients' community reintegration level.

**Methods:**

Post-stroke patients who completed the ICF-PSRP participated in intake and pre-discharge individual face-to-face semi-structured interviews. In addition, case therapists were invited to a face-to-face semi-structured group interview. Clinician experts were invited to complete an interview with the same interview contents as case therapists but in an online format. All interview recordings were analyzed with the Framework analysis. Patients' treatment goals were mapped with the ICF Core Set for Stroke.

**Results:**

Out of 37 invited post-stroke patients, thirty-three of them completed the interview. Three case therapists and five clinicians completed the interviews. The goals set by the patients and their caregivers showed a broadening of their scope over the course of the program. The changes in scope ranged from the activities to the participation and environmental components. Increases in patient-therapist interactions played an essential role in the goal-setting process, which were integral to personalizing the treatment content. These characteristics were perceived by all parties who contributed to the program outcomes.

**Conclusion:**

The application of ICF's principles and core components offers a useful framework for enhancing post-stroke patients' community reintegration level. Future studies should explore the way in which patient-therapist interaction, exposure to environmental factors, and personalized interventions maximize the benefits of applying this framework to the community integration of post-stroke patients.

## 1. Introduction

Stroke leads to cognitive and physical impairments, and thus limitations in daily living functioning (1, 2). Participation and community reintegration are the key outcomes of post-stroke rehabilitation. A patient-centered approach has been widely adopted in post-stroke rehabilitation to enhance rehabilitation outcomes (3). Personalized treatment programs are characterized by goal-setting and intervention planning (4), as well as enhanced patient-therapist interactions (5, 6).

The International Classification of Functioning, Disability, and Health (ICF) conceptualizes an individual's functioning and disability (7). Within the framework, the body functions (ICF-BF) and structures (ICF-BS), activities, and participation comprise of individual's functioning. These components are influenced by two contextual factors: environment and personal characteristics. The framework further stipulates the dynamic interactions between the health problem, the functioning components, and the contextual factors within an individual. The ICF is applied in different disorders in terms of ICF categories to describe an individual's conditions with health-related codes. To facilitate the practical purpose of ICF, condition-specific ICF Core Sets were built by linking specific health conditions with appropriate ICF categories (8). The framework has been applied in clinical programs for patients with mental (9) or neurological disorders (10). Lexell and Brogårdh (11) proposed the use of the ICF to guide patient assessment, goal-setting, training, and outcome measures in neurorehabilitation. One previous study utilized the ICF to conduct assessments and deliver rehabilitation services to children with cerebral palsy and found that it was able to promote patient-centered care and identify barriers and facilitators for improving the children's functioning (12). Only one study was identified so far which examined the benefits of post-stroke rehabilitation incorporating with the ICF (13). The aim of this study was to understand how the integration of the ICF into the delivery of a post-stroke rehabilitation would promote community reintegration.

Although the ICF seems feasible for enhancing community reintegration, previous studies have revealed issues that may hinder its application, such as therapists being unfamiliar with the terminology used to describe patients' health problems with the health and health-related domains in ICF (14), no consensus on the definitions of “activities” and “participation” (15), and no consideration of patients' motivation for prognosis or understanding of the condition (16). These issues may impact the effectiveness of the ICF in post-stroke rehabilitation.

A review of the literature indicated that only a few studies have used the ICF concepts to frame the delivery of post-stroke rehabilitation. For example, a case study of an ICF-based program for a middle-aged post-stroke patient reported improvement in walking abilities and, hence, increased participation in the community (17). Some studies explored the feasibility of using the ICF stroke Core Set to assess post-stroke patient function (18, 19) or to map the contents of the intervention (20). The results confirmed that the ICF was useful for evaluating patients' situations and for representing the training components of the interventions. An earlier study examined the outcomes of a post-stroke intervention based on the ICF, and changes in ICF-BF further enhanced patients’ activities and participation (ICF-A&P) level (21). A recent clinical trial reported that the positive effects of the physical and social environment contributed to the treatment outcomes of post-stroke patients (22). The physical environment in that study comprised the environments requiring physical movements, such as personal activities of daily living, while the social environment included activities in which patients interact with others, such as using body gestures to deliver messages. Thus, we are interested in the application of the framework in outpatient post-stroke rehabilitation and the factors that facilitate its implementation.

This study had two aims. First, we explored the extent to which the ICF can be applied to a post-stroke rehabilitation program for promoting community reintegration in patients via implementation of the ICF-based post-stroke rehabilitation program (ICF-PSRP). Second, we investigated the processes which might contribute to the treatment outcomes of the ICF-PSRP. We hypothesized that the ICF could broaden the scope of assessment and treatment of post-stroke patients particularly in the participation and environmental factors (EF). A personalized approach and patient-therapist-environment interactions enhanced by the ICF would enhance the community reintegration outcomes of post-stroke patients.

## 2. Materials and methods

### 2.1. Participants

Three groups of participants were included in the study. The first group included patients who joined the ICF-PSRP at a rehabilitation centre operated by a non-governmental organization. The inclusion criteria for the patient group were as follows: (1) a diagnosis of stroke with an onset of no more than 24 months, (2) medically stable, (3) able to transfer or walk with no more than one item of assistance, and (4) able to tolerate at least 2 h of active rehabilitation treatment, (5) able to verbally express themselves, and (6) able to understand the interview questions. The types of strokes and number of incident were not limited in this study. The clinical staff group comprised the clinical staff in the rehabilitation centre who were involved in delivering the program to the patients. The clinical expert group were clinical experts who were not involved in the design or delivery of the program, but who were knowledgeable in post-stroke rehabilitation and the ICF. All participants voluntarily participated in the study and provided informed consent before attending the interview. This study followed the Declaration of Helsinki and was approved by the Human Subjects Ethics Sub-committee of Hong Kong Polytechnic University (HSEARS 20210407006).

### 2.2. Study design

This study employed a cross-sectional design using data collected in semi-structured interviews. The guiding interview questions were constructed based on the normalization process theory (NPT) of implementation science. NPT explains the processes by which an individual responds and adapts to a treatment program, such as the four engagement processes: coherence (i.e., consistency between the planned aims and contents of a new intervention and the actual understanding in individuals), cognitive participation (i.e., an individual's psychological preparation to engage, commit and sustain a new intervention), collection actions (i.e., works to do to enact a new intervention), and reflective monitoring (i.e., evaluation which individuals do to assess and understand how a new intervention affect themselves and other individuals) (23, 24). The NPT model has been used in previous qualitative studies to identify the strengths and knowledge of program implementation in hand and arm exercise programs for post-stroke patients (25).

### 2.3. Setting and intervention

The ICF-PSRP aimed to facilitate functional improvement in post-stroke patients, the community, and social reintegration. Patients (and their caregivers, if any) decided on their own to join the program directly after their inpatient stay, parallel or after the outpatient rehabilitation services provided by the hospitals. The intervention program comprised 30–48 sessions (1.5–2.5 h each) over a period of 8–12 weeks, depending on the needs and progress of the patients. The goal-setting, intervention contents, pathways and flow, and assessments were based on the Core Set for Stroke (ICF-CS) for Stroke as the framework (8, 17) and no additional categories in the ICF were included.

**Figure 1:**
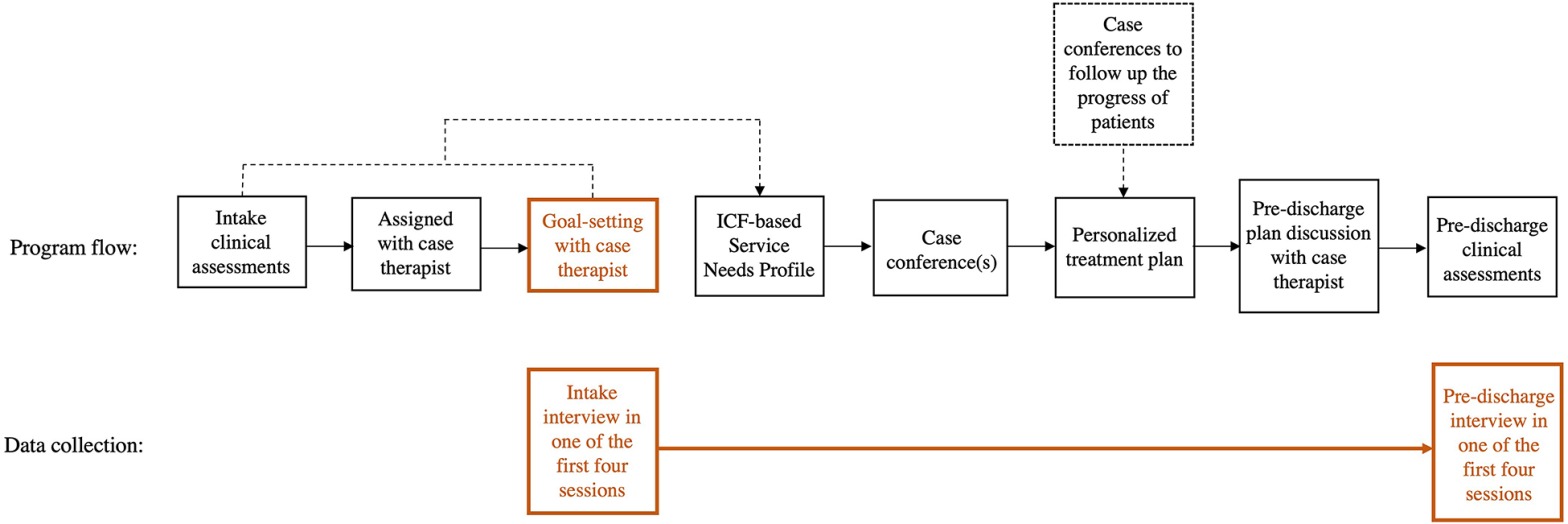
Conceptual illustration of the ICF-based post-stroke rehabilitation program (ICF-PSRP). The first row indicated the program flow from intake clinical assessments to pre-discharge assessments. The ICF was embedded throughout the process. Contents of patients’ goals from the goal-setting process with case therapists highlighted in orange were collected for this study. The second row indicated the intake and pre-discharge interview processes in accordance with the program flow.

After admission, the patients completed intake clinical assessments with therapists and goal-setting with their case therapist (Figure 1). The patients (and their caregivers, if any) discussed their treatment goal(s) with the case therapist. The therapist assisted the patient with setting goals that were related to the ICF-A&P rather than the ICF-BF, and to their life roles and functional gaps. At the case conference, the multidisciplinary program team composed a personalized treatment plan for the patient based on the results of the intake clinical assessments and goal-setting. The progress made by each patient in terms of assessment results and updates to the treatment plans or discharge plans were discussed in monthly case conferences.

The treatment program was designed by a multidisciplinary team with expertise in physiotherapy (PT), occupational therapy (OT), and speech therapy. Each discipline set its treatment aims and developed its specific training contents, intensities, durations, and upgrading and completion criteria to meet patients' goal(s). Treatment contents were comprised of the ICF-BF or ICF-A&P components, and each component was made up of eight treatment modules respectively. For example, PT included strengthening exercises for ICF-BF modules, and gait training for ICF-A&P modules. OT included strengthening exercises for ICF-BF modules, and self-care training for ICF-A&P modules. ST included oral-motor training for ICF-BF modules, and communication training for ICF-A&P modules. The community training module was a multidisciplinary ICF-A&P module on activities in daily routines such as transportation and shopping training.

### 2.4. Data collection procedures

Individual face-to-face semi-structure interviews with the patients (and their caregivers). A group interview with the clinical staff were performed in a quiet room at the rehabilitation centre. Group interviews with experts were conducted using Zoom software. The first author (MW) of this study conducted all the interviews and focus groups. The intake and pre-discharge interviews with the patients were completed within the first and last four sessions, respectively. The interview with therapists was conducted after they had completed handling at least several post-stroke cases in the ICF rehabilitation program. Interviews with experts were conducted in the same period as the interview with therapists.

The leading questions focused on how the design and implementation of the ICF-PSRP were consistent with the concepts and content stipulated in the ICF. For post-stroke patients, there were five and nine questions in the intake and pre-discharge interviews, respectively (Supplementary Table S1 and S2). The flow of the intake interviews was as follows: (1) patients were encouraged to describe their goal(s) and share program-related experience; (2) the interviewer introduced the ICF to patients in layman terms; and (3) patients were asked to describe their motivation to engage in the program. The flow of the discharge interview was as follows: (1) patients were asked to describe the extent to which their goals were achieved and (2) patients were asked to recall the factors that contributed to the program outcomes. All interviews were audio-recorded with the consent of the patients and their caregivers if present. Five guiding questions were used for the clinical staff interviews (Supplementary Table S3). The flow of the staff interview was as follows: (1) staff were asked to compare the ICF-PSRP with other conventional programs and (2) were then asked to suggest continuous improvements in the program. The interviews with clinical experts used the same set of guiding questions (Supplementary Table S3). However, a set of information about the flow and design of the ICF-PSRP was given to the clinical expert group beforehand to get familiarize with the rehabilitation program. The interviews with both the clinical staff and experts were audio-recorded with informed consent.

### 2.5. Data analysis

Demographic variables were descriptively analyzed for the intake and the pre-discharge group.

Interview recordings were analyzed using a qualitative framework analysis, while patient goals mentioned during interviews were linked with the ICF-CS.

#### 2.5.1. Framework analysis

All Cantonese interview recordings were transcribed verbatim by MW using iFLYREC (26), and the contents were compared with those transcribed by another researcher. Transcribed data were mapped on the theme framework in five steps (27, 28): Step 1: data familiarization––MW and another researcher (HT) read the interview transcripts and field notes to develop an overview of themes; Step 2: thematic framework construction––MW and HT independently identified themes and sub-themes based on similarities in concepts and relationships among the concepts, constructed theme and sub-theme definitions and names by consensus, and tested against two transcripts; Step 3: indexing––transcript contents consistent with the theme/sub-theme framework were coded, and new themes and subthemes were created in the course of the analysis (Supplementary Table S4); the refinement process was terminated once MW and HT found data saturation; Step 4: charting––**s**ummarized data for each participant were placed in a row and themes were placed in columns (28), in addition, a comment column containing verbatim quotes or codes for each participant was added; and Step 5: mapping and interpretation––conclusions, patterns and structures of the themes and sub-themes were identified and cross-checked against the original data, and the relationships among the themes were abstracted and interpreted.

#### 2.5.2. Mapping with ICF-Cs

The rules for mapping patients' goal contents to the ICF-CS for Stroke were based on ICF linking rules stipulated by Cieza et al. (29) and Fayed et al. (30). Contents with similar concepts were grouped into the ICF domains (31), which were then linked to the ICF categories at different levels.

## 3. Results

### 3.1. Participant information and demographic variables

Thirty seven post-stroke patients were invited to join the interviews. A total of 33 post-stroke patients participated in the study. Twenty-six patients involved in the intake interview (i.e., 26 focus group intake interviews), and 26 patients involved in the pre-discharge interview (i.e., 26 focus group pre-discharge interviews). Nineteen participants joined both intake and pre-discharge interviews. The remaining participants participated in either the intake (*n* = 7) or the pre-discharge interview (*n* = 7). Each interview lasted for 20–50 min, depending on the conditions and needs of the patients. Demographic variables of patients were indicated in Table 1. In the intake interview, ten patients reported having an ischemic stroke, and 12 reported having a hemorrhagic stroke. One patient reported having an ischemic stroke followed by a hemorrhagic complication, and three patients did not indicate their type of stroke. Seventeen patients (65.4%) had left hemiplegia, 8 patients (30.8%) had right hemiplegia, and one patient had diplegia. In the pre-discharge interview, 12 patients reported having an ischemic stroke, and another 12 reported having a hemorrhagic stroke, and two patients did not indicate their type of stroke. Fourteen patients (53.8%) had left hemiplegia, 11 patients (42.3%) had right hemiplegia, and one patient had diplegia.

**Table 1:** Demographic characteristics of the post-stroke patient participants in the intake and pre-discharge interviews.

Variables	*N* (%) in intake interview	*N* (%) in pre-discharge interview
Age range (in years)	44–73	36–73
Mean age (in years; *SD*)	55.15 (7.49)	57.04 (9.57)
Gender (*SD*)		
Male	21 (80.8)	17 (65.4)
Female	5 (19.2)	9 (34.6)
Types of stroke		
Ischemic stroke	10 (38.5)	12 (46.2)
Hemorrhagic stroke	12 (46.2)	12 (46.2)
Ischemic stroke with hemorrhagic complication	1 (3.8)	0 (0)
Not indicated	3 (11.5)	2 (7.7)
Months since stroke* (*SD*)	8.08 (6.25)	7.69 (6.35)
Side of hemiplegia		
Left	17 (65.4)	14 (53.8)
Right	8 (30.8)	11 (42.3)
Bilateral	1 (3.8)	1 (3.8)

SD, standard deviation.

* Calculated between the date of the stroke to the date admitting to the program.

Clinician participants were involved in one group interview. They comprised one physiotherapist, one occupational therapist, and one speech therapist. Five other clinical experts were recruited to form the clinical expert group. Two group interviews were completed. The first interview consisted of one professor from a local university with expertise in public health, a medical doctor who is a consultant in rehabilitation, and a patient expert who was a stroke survivor with expertise in rehabilitation service development and policy. The second interview consisted of another professor from a local university with expertise in public health, and an occupational therapist involved in stroke rehabilitation. Each interview for the clinical staff and clinical expert groups lasted for 60 min.

The themes and sub-themes identified based on the participants' responses in the face-to-face interviews were organized according to the intake and pre-discharge occasions. Nineteen participants joined both intake and pre-discharge interviews. There were four themes: patients' goal-setting, patients' interactions with therapists and the environment, ICF content domains, and ICF-based interventions. Responses obtained from the staff and clinical experts are presented where appropriate.

### 3.2. Theme 1 (intake): patients' goal-setting

#### 3.2.1. Sub-theme 1.1: rehabilitation goals proposed by patients

Ten patients set four goals (38.5%), eight set three goals (30.8%), four set five goals (15.4%), three set two goals (11.5%), and one patient set one goal (3.8%). Among them, “improving walking ability” was the first-ranked goal set by most patients (*n* = 19). The targets set for this goal included walking with a quadruped stick without assistants, a decrease in the number of assistants, and walking posture and endurance.

# 27 (from his sister): “*Brother you answered, “I hope I can walk,” but the therapist said, ‘You now…need two assistants to support you. Hope one assistant can support you after this treatment.’”*

The second-ranked goal was “resuming work” (*n* = 14).

# 33: “*My goal is to wish for resuming work…I think most stroke patients share the same thought…Hope to resume work as quickly as possible…to earn money as the breadwinner.”*

The third- and fourth-ranked goals were “improving upper limb function” (*n* = 12) and “self-care ability” (*n* = 11), respectively.

#### 3.2.2. Sub-theme 1.2: previous experiences influenced goal-setting

Most of the patients (*n* = 24) had been discharged from public hospitals, while others (*n* = 12) had received outpatient rehabilitation at hospitals or outpatient clinics. According to them, they had not been asked to set goals, and treatment programs focused on functional deficits.

# 30: “*[The hospitals] seldom talk about goals… When they [therapists] observe you are not able to walk… you need to be trained on walking.”*

### 3.3. Theme 2 (intake): interaction with therapists and environments

#### 3.3.1. Sub-theme 2.1: patient-therapist interaction in goal-setting

At intake, as part of the program protocol, case therapists would coach the patients to set treatment goals. As most patients were not familiar with setting goals, case therapists focused on explaining the procedures and the reasons behind the exercise. The patients tended to set few goals (i.e., one to two), and the contents and targets seemed to be bound by the duration of the current program.

# 31: “*[The therapist] asked what I want to improve…I am not sure … see if I can…with the aid of exercises or other [assistance]…to enhance my abilities.”*

From the therapist's perspective, setting goals for treatment planning with patients was a new approach adopted by the rehabilitation center. They found that the process involved more interactions with patients than conventional practices. They reported that patients seemed to benefit from the goal-setting process and had a clearer mindset about their engagement in treatment sessions. From the experts' perspective, the goal-setting stage promoted patients' active participation. The experts further asserted that goal-setting would enable common ground between patients and therapists.

#### 3.3.2. Sub-theme 2.2: patient-environment interactions

Most goals set by the patients were not environment specific but could be achieved within the rehabilitation center or their own home. Common goals were to regain walking ability (e.g., # 30), upper limb functions (e.g., # 7), and self-maintenance ability at home (e.g., # 17).

# 30: “*[I wish] I can walk [by myself] … [currently when I walk] from the bedroom to the living room, and walking to the toilet needs to rely on … my wife and domestic helper to hold me.”*

Therapists expressed that the goal-setting process would be a good opportunity to encourage patients to consider the roles of the environment in restoring their life and social roles. They shared that they had tried to institute this at their intake.

### 3.4. Theme 3 (intake): ICF content domains

The participants (*n* = 26) set 94 intake goals. The majority of the goals (94.7%) were classified under the ICF-A&P component (Table 2A). The remaining goals (5.3%) were classified according to the ICF-BF and ICF-BS components. Nearly half of these goals (45.4%) included at least one EF, while a smaller proportion of the goals (14.9%) were related to self-care activities. One individualized treatment goal could be linked to more than one ICF categories.

**Table 2A:** Frequency counts and ICF-CS categorization of the goals set (*n* = 63) by more than 4% of the participants at the intake interview.

Goals in intake interview	Frequency (%)	Activities/Participation/Environmental factors
Improving walking ability	19 (20.2%)	d450 Walking, d460 Moving around in different locations, d465 Moving around using equipment
Resuming work	14 (14.8%)	d845 Acquiring, keeping, and terminating a job, d850 Remunerative employment, d855 Non-remunerative employment e135 Products and technology for employment
Self-care ability	11 (11.7%)	d510 Washing oneself, d530 Toileting, d540 Dressing, d550 Eating e115 Products and technology for personal use in daily living
Improving upper limb function	10 (10.6%)	d430 Lifting and carrying objects, d440 Fine hand use, d445 Hand and arm use
Taking care of family	5 (5.3%)	d570 Looking after one's health, d620 Acquisition of goods and services, d630 Preparing meals, d640 Doing housework
Driving	4 (4.3%)	d475 Driving e540 Transportation services, systems, and policies

Codes begin with “d” equivalent to activities and participant categories; codes begin with “e” equivalent to environmental factors; No goals cover body functions categories in the table.

### 3.5. Theme 4 (intake): ICF-based intervention contents

The participants would not have gained enough experience to reflect on the treatment received, as all interviews were conducted within the first four sessions. However, the content formed the basis for meaningful comparisons with those gathered at the pre-discharge occasion. One participant (# 12) shared his experience gained from attending the “community training module” using an escalator. He commented that the approach taken by the therapist (standing next to him) increased his confidence in performing the new task. Other participants commented that the training modules of the program were relevant to their goals and shared that this training was useful for functional regain.

# 33: “*I told the therapist that … I need to climb the ladder during work … then she [the therapist said] let us try climbing a few steps … the therapist knew my goal.”*

### 3.6. Theme 1 (pre-discharge): patients' goal-setting

Goal-setting was conducted between therapists and patients during the program. Thirty-seven goals were set by 26 patients who participated in the pre-discharge data collection; 11 patients (42.3%) reported that they set two goals, and 15 (57.7%) reported that they set one goal.

#### 3.6.1. Sub-theme 1.1: rehabilitation goals proposed by patients

Similar to the intake interviews, “improving walking ability” remained the most common goal set by the patients (*n* = 14). Contrariwise to the intake interview, the contents of this goal became more specific, such as increasing endurance and walking without accessibility.

# 4: “*Hope…after training…I can…control myself [walking ability] when going out … like grocery shopping, is still a problem now.”*

The specificity of the “improving upper limb function” goal, which ranked second in problem setting, was the use of chopsticks and spoons in eating, writing at work, and carrying items while shopping.

#### 3.6.2. Sub-theme 1.2: goal accomplishment in the program

Among the 37 goals set by patients, 30 experienced moderate-to-large improvements as commented by the patients. Fourteen patients reflected their “walking ability” improved in terms of speed, endurance, and posture. Four patients evaluated improvements in “upper limb function.” In particular, one patient added that she could begin performing housework, including cooking, one month into the program. Three patients reported improved dressing and toileting ability. One patient shared his progress toward the “resuming work” goal with improvements in physical functions.

### 3.7. Theme 2 (pre-discharge): interaction with therapists and environments

#### 3.7.1. Sub-theme 2.1: patient-therapist interaction in goal-setting

Patients described their interactions with case therapists, such as setting short- and long-term treatment goals. For instance, one patient (# 1) explained in detail how an occupational therapist worked with her improvement levels and timelines of treatment upgrades. Therapists consistently expressed the importance of embedding activities rather than body functions training in treatment programs. They also shared their skills to help patients attend to their daily living needs and ask them the “why” questions.

Therapist # 1: “*I provide them with more ways to think … and then drill them to write concrete goals … [the patient] wanted to walk longer and stronger … Why? Any curbs to cross? Any slope to climb?”*

#### 3.7.2. Sub-theme 2.2: patient-therapist interaction in training contents

Twenty-four patients managed to recall the details of the interactions with the therapists. They included follow-up actions taken on assistive equipment, remediation of walking posture, modifying and upgrading treatment contents, and discussions about pre-discharge plans. Patients expressed that they found the therapists helpful and responsible, while the therapists shared that they actively attended to the patients' feedback and opinions, especially those on the effects of treatment, to address their goals.

#### 3.7.3. Sub-theme 2.3: patient-environment interactions

Sixteen patients reported their (or caregivers') experiences interacting with different physical environments. Examples of the environment mentioned included window shopping at a supermarket near the home and dining out in a restaurant after taking the Mass Transit Railway (underground train). Thirteen patients described their interactions with the social environment within or outside the rehabilitation center. The social environment mentioned involved other patients in the same post-stroke program for exchange of therapy-related information, meeting co-workers at the workplace, and seeing family members and friends in social gatherings outside the home.

### 3.8. Theme 3 (pre-discharge): ICF content domains

Twenty-eight goals (75.7%) were classified under EF compared with 44 goals (94 goals; 45.4%) classified in the intake interview (see Table 2B). In addition, the goals set in the pre-discharge interviews were contextualized by participation and EF rather than by activities components (Table 3). For example, “walking to Chinese restaurant to yum cha” might relate to “e150 Design, construction and building products and technology of buildings for public use” of the restaurant and gathering of “e315 Extended family” or “e320 Friends.”

**Table 2B:** Frequency counts and ICF-CS for stroke categorization of the goals set (*n* = 10) by more than 2% of the participants and some examples at the pre-discharge interview.

Goals in pre-discharge interview	Frequency (%)	Activities/Participation/Environmental factors
Walking and shopping	4 (10.81%)	d450 Walking, d460, Moving around in different locations, d620 Acquisition of goods and services e150 Design, construction, and building products and technology of buildings for public use
Taking Mass Transit Railway	3 (8.11%)	d470 Using transportation e540 Transportation services, systems, and policies
Walking to Chinese restaurant to yum cha	2 (5.41%)	d450 Walking, d460, Moving around in different locations, d910 Community life e150 Design, construction and building products and technology of buildings for public use, e310 Immediate family, e315 Extended family, e320 Friends
Computer typing to assist children's homework	1 (2.70%)	d440 Fine hand use e115 Products and technology for personal use in daily living
Using chopstick	1 (2.70%)	d440 Fine hand use, d550 Eating e110 Products or substances for personal consumption

The goals indicated in the table are specific to patient needs. Patients with more severe sequelae who set goals that were non-specific to any context (i.e., not involving any environmental factors, and there were eight in total) were not indicated here. Codes begin with “d” equivalent to activities and participant categories; codes begin with “e” equivalent to environmental factors; No goals cover body functions categories in the table.

**Table 3:** Comparisons of patients’ goals set during the intake vs. pre-discharge interviews.

Goals in intake interview	Goals in pre-discharge interview
Improving walking ability	Walking to Chinese restaurant to yum cha Shopping and taking escalator in a shopping mall Stair walking Taking escalator
Taking transportation	Taking Mass Transit Railway Taking bus Taking minibus
Improving upper limb functioning	Writing in workplace Using chopstick Using hand to carry items in shopping

### 3.9. Theme 4 (pre-discharge): ICF-based intervention contents

Both patients and experts commented on the new contents of the ICF-PSRP. They attributed this new content to the adoption of the ICF to design the program's flow of service. The goal-setting process embedded with ICF concepts seemed to drive a personalized approach to program delivery.

#### 3.9.1. Sub-theme 4.1: community training

Eight patients reported receiving community-based training. Six of them commented that the program, delivered in the community, would facilitate their return to their accustomed environment. For example, one patient (# 28) received training with a therapist using an escalator at the Mass Transit Railway station near the rehabilitation centre. He further explained that he was now able to manage the escalator, which he had previously perceived as too fast to handle.

#### 3.9.2. Sub-theme 4.2: personalized training contents

Ten patients considered the training modules to be specially designed to meet their set goals and treatment needs. They found that these modules occupied a larger proportion of the program, which differed from the rehabilitation services they received. Examples of these modules were grocery shopping, which involved walking endurance training, and handwriting at work, which involved fine hand function training. The therapist also shared that the patients had a goal-directed design for the intervention program. The content of the modules needed to be specific according to the context and targets of the goals. This approach differs from a conventional program in which the interventions are standardized and general in nature. Therefore, the ICF was found to be helpful in defining participants' needs for participation and their interactions with the physical and social environment. The experts' views were similar to those of the therapists.

Expert # 1: “*With the ICF framework, we already had a holistic approach. We can see broader … on the patients’ needs. From day one [of the rehabilitation], we can … orientate the training toward … his longer-term goals.”*

## 4. Discussion

This study examined the application of the ICF to post-stroke rehabilitation program for enhancing patients' community reintegration. From the patients' and therapists' perspectives, we interpreted that the ICF enhanced the outcomes of the rehabilitation program by strengthening patients' participation and by emphasizing the impacts of EF. The goal-setting and patient-therapist interactions embedded in the program design contributed to personalized program delivery for post-stroke patients.

Our results indicate that implementing the goal-setting process facilitated program outcomes. The interactions between the patients and their case therapists throughout the goal-setting process seem to be crucial for an effective personalized program. More than half of the goals (61%) set by the patients in the intake interviews were related to ICF-BF and activity components. Approximately 30 percent of the goals were related to these two components. Our findings are consistent with those of a previous study on a post-stroke program in which patients' goals formulated at admission concentrated on ICF-BF and activity-based improvements, such as walking ability and upper limb functions (32). In this study, the interview scripts indicated that the contents of the patients' goals were largely influenced by the service provision at the acute stage or hospital-based intervention programs. This finding concurred with a study on conventional rehabilitation programs, suggesting that patients' experiences were predetermined by therapists and standardized training content (33). These goals were inclined to be impaired and less applicable to patients' community reintegration needs. In contrast, the results of the pre-discharge interviews in this study indicated that patients' goals tended to be geared toward their unique life situations, environment, and desires for participation. For instance, patients expressed the need to use an escalator independently instead of merely improving their walking ability. The number of goals classified into the participation component increased from 23 percent in the intake to 62 percent in the pre-discharge interviews. The changes in the content of the goals set by the patients who joined the program between admission and pre-discharge reflected the positive effects of patient-therapist interactions in program delivery, particularly goal-setting. Our results further indicate that the case therapists helped patients explore goals and set targets to achieve them. Agreed goals between patients and therapists were specific in nature and aimed at solving short-term problems faced by patients (34). The built-in content flexibility of treatment plans according to patients' goals contributed tremendously to personalized intervention for this program. The benefit of patients actively participating in goal-setting is enhanced program outcomes (32, 35). More importantly, the goal-setting process and relevance of the training content to the goal can effectively improve patients' treatment engagement and adherence (36). Patients who realized their treatment and training goals showed higher engagement in training intensity than those who only received non-specific training instructions.

The importance of the patient-therapist interaction in the program was highlighted by having both the patients and therapists participate in this study. Both groups commented that interactions increased as the sessions progressed. Two observations were made. First, at intake, the patients' mindsets appeared to be dominated by their previous experiences in hospital-based rehabilitation programs. The majority of the patients interviewed displayed reserved goal-setting and hesitated to play an active role in interacting with the case therapists. This result is consistent with existing literature stating that patient behaviors may result from perceived professional dominance in rehabilitation (37, 38). Second, we observed the benefits of personalized treatment offered by the program and one-on-one support from case therapists. The patient-therapist interaction was the key to bringing about personalized training content designed according to the patients’ goals. Patients expressed their concerns about their needs and progress to their case therapists––an opportunity denied in conventional settings. These interactions contributed to the positive experiences of care and satisfaction reported by our patients. Patients perceive the amount and quality of patient-therapist interactions as more important than the amount and content of treatment (39). A sufficient number of interactions was considered by patients as valuable content in rehabilitation, which enhanced treatment outcomes (3).

The assessment and treatment of the ICF-PSRP were based on the ICF. One aim of the program was to expose patients to the EF of the ICF, such as how technology and policies facilitate or hinder community reintegration. During the intake interviews, patients tended to perceive their ICF-BF and activity-based deficits as obstacles to dependent living. They further explained that the EF had been less of a concern as their environment had centered around the home and rehabilitation center. They did not perceive the need to go elsewhere, and their caregivers could help whenever necessary. In the pre-discharge interviews, there was a drastic increase in the proportion of patients who expressed concerns about their limitations from an environmental context. A significant reason, as expressed by both the patients and therapists, was the living environment exposure during training. Participants often window-shopped in supermarkets and took escalators in the Mass Transit Railway during training. Our findings are consistent with those of a previous study that emphasized the EF embedded in post-stroke rehabilitation (40). The results indicated the effect of conducting training in environments similar to real-life situations on mobility in the community. Other studies have reported the application of EF in treatment programs (41, 42). For example, Debrouwere et al. (41) used ICF-based patient profiles to delineate patient health conditions based on the ICF components. The framework demonstrated sensitivity in identifying patients' situations and factors that contribute to treatment, including personalized EF.

The findings of this study demonstrate the importance of patient-therapist relationships in enhancing treatment outcomes. The ICF-PSRP focus on nurturing positive patient-therapist relationships at the start of the program during the goal-setting process. Goal-setting guided by the ICF-based content, particularly participation and EF, would help patients and therapists target independent living. These goals should form the basis for delivering personalized treatment programs for patients. Service providers, however, should be aware of possible increases in human and nonhuman resources. The anticipated increase in resources would be prominent for settings that lack experience implementing the patient-centered approach in service delivery. Clinicians are likely to invest time during the intake interview to familiarize patients discharged from acute settings with the goal-setting process and ICF content domains.

## 5. Conclusion

The current study examined the extent to which the ICF can be applied to an ICF-PSRP and the significant factors that would enhance the program's outcomes. Using a qualitative study design, the results indicate that patient/case therapist consensus goal-setting, strong patient-therapist relationships, and personalized intervention are significant factors contributing to program outcomes. The ICF is vital in offering a wider scope of concerns for patients and therapists when formulating a treatment plan other than improvements of their body functions and structures. The patient-therapist and environment interactions embedded in personalized interventions have been found to shift patients' concerns from ICF-BF to ICF-A&P and EF. Further studies are needed to reveal the effectiveness and cost-benefit of an ICF-PSRP for post-stroke patients.

## Data Availability

The datasets presented in this article are not readily available because patient's identities may be revealed from the original interview scripts. Requests to access the datasets should be directed to ngai-kiu.wong@connect.polyu.hk.
