# Supplementary material for: Goal-setting and personalization under the International Classification of Functioning, Disability, and Health framework: Community reintegration program for post-stroke patients
Source: Front Rehabil Sci. 2023 Aug 4;4:1219662. doi: 10.3389/fresc.2023.1219662 (PMC10436562; doi:10.3389/fresc.2023.1219662)
Supplement: Supplementary file 1 [file Table1.docx]

**Supplementary Materials**

*Table S1. Guiding questions in intake and pre-discharge patient interviews.*

| Guiding questions^#^ |
| --- |
| 1. What are your goals to achieve in this treatment program? 2. Could you provide details of previous treatment program(s) that you joined?^*^ 3. When compared with the program you joined before, how different in terms of the aims do you think about this treatment program and the one you joined? 4. When compared with the program you joined before, how different in terms of the contents do you think about this treatment program and the one you joined? 5. You know that this treatment program is different from your previous treatment program, do you believe that joining this program can help you achieve your goals and return to the community as much as possible? |

^#^Questions 1, 3 to 5 were asked in both intake and pre-discharge interviews to observe patients’ perceived changes after joining the ICF-PSRP.

^*^Question 2 was asked in the intake interview.

*Table S2. Guiding questions in pre-discharge patient interview.*

| Guiding questions |
| --- |
| 1. Did you observe improvements in general on yourself throughout the treatment program? What improvements on yourself did you observe? 2. Given your improvements from the treatment program, did you think these improvements can also fulfil the goals you set at the beginning? 3. In what aspects do you think this treatment program is good for other individuals who have similar problems with you? 4. In what aspects do you think the treatment program need to change to achieve your goals? |

*Table S3. Guiding questions in focus groups.*

| Guiding questions |
| --- |
| 1. [Open-ended question] When compared with a conventional program, how different in terms of the treatment aim do you think this ICF program is? Specify those differences. 2. [Open-ended question] When compared with a conventional program, how different in terms of the treatment contents do you think this ICF program is? Specify those differences. 3. [Open-ended question] What other features in this ICF program do you think are important in addition to the unique assessment and treatment contents? 4. [Open-ended question] Are there any other specific issues or concerns you think in implementing the ICF against post-stroke rehabilitation? 5. [Open-ended question] Are there any actions that therapists in this program can do to enhance the service outcomes with the ICF? |

*Table S4. Final Thematic Framework for Indexing and Charting*

| Themes | Definitions | Sub-themes | Definitions |
| --- | --- | --- | --- |
| Rehabilitation goal(s) | Step(s) to tailor rehabilitation to meet patients’ needs, which is a behavior change technique to enhance clinical outcome(s) from intervention(s) (1) | Shorter-term goal(s) | The intended consequence of actions defined by rehabilitation team and patient (2) |
|  |  | Longer-term goal(s) | Patients’ indented future state, include their hopes and aspirations (1, 2) |
| Perceived content | Set of previous interventions designed to optimize functioning and reduce disability in post-stroke patients to interaction with their environment (3) | Other experiences | Rehabilitation experiences received other than the current program (e.g., from public services or private sectors) |
| Perceived goal | Rehabilitation goal(s) set in different services to optimize functioning and reduce disability in post-stroke patients to interaction with their environment (3) | Current experiences | Rehabilitation experiences expected or received in the current program |
| Perceived personnel | Staff, including therapists in different disciplines and rehabilitation assistants, involved in the rehabilitation process (4) | Comparison | Comparison of [theme] between other programs and the current program |
| Evaluation of self in rehabilitation program | Patients’ contribution and rehabilitation outcome perception | Goal-based outcome comparison | Individualized outcome achievement from the program (5) |
| Evaluation of rehabilitation program | Factors and patients’ opinions that may affect rehabilitation outcome(s) | / | / |
| Therapeutic relationship | Collaboration between patients and therapists to identify and achieve program-specific and long-term rehabilitation goals. Therapist caring toward patients promotes desired outcomes (6) | Other experiences | Rehabilitation experiences received before the current program (e.g., from public services or private sectors) |
|  |  | Current experiences | Rehabilitation experiences expected or received in the current program |
| Patient-environment interaction | Patients interact with environments inside and outside of the rehabilitation centre, including surrounding objects and people, to restore or return to a state of optimal functioning (7) | Physical environment | Environments can be barriers or facilitators to patients. Changes in the physical environment influence patients’ activity and social interactions (8) |
|  |  | Social environment | Interactions or activities to support patients’ social contact and communication skills with friends, family, and others (9) |
| Lack of stroke rehabilitation knowledge | Patients may show insufficient knowledge on continuing stroke rehabilitation and aspects related to it (10) | / | / |
| Build up confidence from therapists | Positive reinforcement and encouragement received from therapists that helped to build belief in patients (11) | / | / |
| Practice against goal | Patients practiced on their outside the rehabilitation center | / | / |
| Community reintegration | Environments that patients have been apart from their home and rehabilitation centres (12) | / | / |

**References**

1. Dekker J, de Groot V, Ter Steeg AM, Vloothuis J, Holla J, Collette E, et al. Setting meaningful goals in rehabilitation: Rationale and practical tool. Clin Rehabil. 2020;34(1):3-12. doi: 10.1177/0269215519876299

2. Wade DT. Goal setting in rehabilitation: An overview of what, why and how. Clin Rehabil. 2009;23(4):291-5. doi: 10.1177/0269215509103551

3. World Health Organisation. Rehabilitation: Author; 2021, November 10 [cited 2021 November 10]. Available from: https://www.who.int/news-room/fact-sheets/detail/rehabilitation.

4. Christakou A, Lavallee D. Rehabilitation from sports injuries: From theory to practice. Perspect Public Health. 2009;129(3):120-1266. doi: 10.1177/1466424008094802

5. Turner-Stokes L. Goal attainment scaling (GAS) in rehabilitation: A practical guide. Clin Rehabil. 2009;23(4):362-70. doi: 10.1177/0269215508101742

6. Cummins C, Payne D, Kayes NM. Governing neurorehabilitation. Disabil Rehab. 2021;44(17):1-8. doi: 10.1080/09638288.2021.1918771

7. Bonnechère B, Van Sint Jan S. Chapter 39 - Rehabilitation. In: Scataglini S, Paul G, editors. DHM and Posturography: Academic Press; 2019. p. 541-7.

8. Anåker A, von Koch L, Eriksson G, Sjöstrand C, Elf M. The physical environment and multi-professional teamwork in three newly built stroke units. Disabil Rehab. 2020;44(7):1-9. doi: 10.1080/09638288.2020.1793008

9. Kylén M, Ytterberg C, von Koch L, Elf M. How is the environment integrated into post-stroke rehabilitation? A qualitative study among community-dwelling persons with stroke who receive home rehabilitation in Sweden. Health Soc Care Community. 2021;30(5):1933-43. doi: 10.1111/hsc.13572

10. Kamalakannan S, Gudlavalleti Venkata M, Prost A, Natarajan S, Pant H, Chitalurri N, et al. Rehabilitation needs of stroke survivors after discharge from hospital in India. Arch Phys Med Rehabil. 2016;97(9):1526-32.e9. doi: 10.1016/j.apmr.2016.02.008

11. Horne J, Lincoln NB, Preston J, Logan P. What does confidence mean to people who have had a stroke? –A qualitative interview study. Clin Rehabil. 2014;28(11):1125-35. doi: 10.1177/0269215514534086

12. Brookfield K, Mead G. Physical environments and community reintegration post stroke: Qualitative insights from stroke clubs. Disabil Soc. 2016;31(8):1013-29. doi: 10.1080/09687599.2016.1223606
